# The order of multisensory associative sequences is reinstated as context feature during successful recognition

**DOI:** 10.1038/s41598-025-02553-3

**Published:** 2025-05-24

**Authors:** Marike Christiane Maack, Jan Ostrowski, Michael Rose

**Affiliations:** Department of Systems Neuroscience, University Medical Center Hamburg‑Eppendorf, Martinistr. 52, Building W34, 20248 Hamburg, Germany

**Keywords:** Learning and memory, Neuroscience

## Abstract

The ability of the human brain to encode and recognize sequential information from different sensory modalities is key to memory formation. The sequence in which these modalities are presented during encoding critically affects recognition. This study investigates the encoding of sensory modality sequences and its neural impact on recognition using multivariate pattern analysis (MVPA) of oscillatory EEG activity. We examined the reinstatement of multisensory episode-specific sequences in *n* = 32 participants who encoded sound-image associations (e.g., the image of a ship with the sound of a frog). Images and sounds were natural scenes and 2-second real-life sounds, presented sequentially during encoding. During recognition, stimulus pairs were presented simultaneously, and classification was used to test whether the modality sequence order could be decoded as a contextual feature in memory. Oscillatory results identified a distinct neural signature during successful retrieval, associated with the original modality sequence. Furthermore, MVPA successfully decoded neural patterns of different modality sequences, hinting at specific memory traces. These findings suggest that the sequence in which sensory modalities are encoded forms a neural signature, affecting later recognition. This study provides novel insights into the relationship between modality encoding and recognition, with broad implications for cognitive neuroscience and memory research.

## Introduction

The ability to remember episodes from the past is a cornerstone of human memory. Episodes consist of multiple features that may stem from different modalities (e.g., visual, auditory), reaching us in specific sequences. In line, it has been shown that humans encode not only the semantic content but also the temporal order (sequence) of features, which is crucial for recalling the flow of a past episode^1,2^. The capacity to encode and retrieve sequential information allows us to mentally rebuild the dynamic structure of events, highlighting the role of temporal context in episodic memory^1,3^. Recalling the order that features of an episode were originally encoded in, is based on sequential reinstatement^4^.

Episodic memory relies on the integration of contextual information during encoding, with the hippocampus playing a key role in binding event features such as sensory modality and spatial-temporal context^5–7^. Prior research has investigated how we remember the temporal order of events, including the role of unimodal cueing^8–10^. Evidence suggests that episodic memory involves temporal compression and event segmentation, where the hippocampus supports memory organization by structuring event sequences and contextual boundaries^9,11^. Temporal compression refers to the tendency of episodic memory to condense events during recall, influenced by event segmentation and goal-directed actions^12–15^. Event segmentation, in turn, affects how temporal order is remembered, as events chunked at perceptual boundaries enhance object-context binding but may reduce precise temporal order memory^9,16^. The hippocampus further supports the encoding and retrieval of event sequences, integrating spatial and temporal contexts essential for remembering event order^17,18^. In line, episodic memory retrieval is shaped by the availability of contextual information at encoding, with reinstatement of encoding context enhancing recognition in providing characteristic cues that mitigate interference^19–21^. Sensory modality sequences, as part of contextual information, contribute to the organization of memory representations^22,23^. The hippocampus is crucial for integrating these contextual elements, facilitating recognition by reactivating modality-based associations rather than strictly reconstructing event sequences in order^18,24,25^.

While previous studies have explored unimodal cueing and temporal order memory, the current study specifically investigates whether the modality sequence acts as a contextual feature that influences encoding and recognition rather than sequential recall per se. Contextual cues, including sensory modality sequences, can mitigate interference effects and improve memory performance by reinstating elements of the original encoding context^21,26,27^. Conversely, mismatches between encoding and retrieval contexts may lead to competition between overlapping memory traces, impairing recall^4,28,29^. Both physical and mental reinstatement of contextual information facilitate episodic retrieval, with mental reconstruction yielding comparable benefits to direct environmental cues^30,31^. Furthermore, cognitive control plays a role in sustaining contextual reinstatement, as individuals with higher working memory capacity are better able to maintain contextual associations through strategic memory processes^32^.

Beyond unimodal sequences, results from the animal, as well as human studies, suggest that sequential information plays a critical role in recalling multisensory episodes, where encoded features from different sensory modalities (e.g., an image-sound pair) are reinstated during retrieval^33–36^. Multisensory features enable the brain to store and retrieve information across various perceptual domains, enhancing our ability to recall past experiences and make informed decisions^37–39^. As such, the human memory has evolved to function optimally under multisensory conditions^40^. Interestingly, multisensory memories can be cued by unimodal features upon remembering, suggesting that multisensory encoding enhances subsequent recognition^39,41^. This means that one modality may serve as a cue to retrieve the complete episode, even if it includes features from different modalities^42^. It is, however, unclear so far whether and how one modality (i.e., auditory) relates to the reinstatement of another (i.e., visual). The Scene Reconstruction Theory suggests that especially the hippocampus helps to reconstruct memories by integrating various sensory details associated with an event^4^. This integration is supposed to allow richer, and more detailed, memory formation, as the brain can draw upon multiple sensory inputs to create a coherent narrative of the event^11,43^.

While neural plasticity allows multisensory learning^44^, understanding the neural reinstatement of sequential information across sensory modalities remains crucial for elucidating the broader mechanisms of remembering. Recall generally involves the activation and reconstruction of neural pathways tied to previously encoded details^45^. This process is influenced by retrieval cues and familiarity with the material^46^. Beyond sequential and multisensory reinstatement, the recollection of contextual details surrounding past events, such as where and when they occurred, significantly contribute to the liveliness and specificity of the memory representation^47–50^. The Context Maintenance and Retrieval (CMR) model here provides a framework to understand how the brain organizes memories around contextual and temporal cues, facilitating the accurate retrieval of episode-specific feature sequences^51,52^, propelling the reconstruction of the correct order and updating of associations. In line, previous research has highlighted that the process of episodic remembering involves not only recalling specific items but also reinstating the contextual details of the original event^53–55^. These studies have demonstrated that the success of memory retrieval is closely associated with the reactivation of the encoding-related memory trace^52,56–58^. Interestingly, it has been shown that during memory reactivation, not only episode-specific features but also contextual features, that were not directly related to the current memory task, are reinstated^29,59–65^. However, the precise neural mechanisms underlying this sequential reinstatement, especially in multisensory contexts, remain elusive.

EEG is a powerful tool to track these reinstatement processes including multisensory episode-specific feature sequences. Here, especially multivariate temporal-pattern analysis has emerged as the gold-standard to examine how neural activity during retrieval reflects (sequential) reinstatement. Moreover, it represents a tool for investigating the role of context reinstatement in memory processes, revealing the (beneficial) effects of reinstating neural encoding patterns in memory retrieval^30,64^. Accordingly, multivariate pattern analysis (MVPA) has been used to decode oscillatory activity patterns during memory retrieval, successfully classifying specific neural signatures tied to remembering^30,64^. Here, low-frequency activity (e.g., beta (13–30 Hz) and theta (3–7 Hz) oscillations) have been shown to be particularly important for episodic memory processes, facilitating successful retrieval^66–70^. Multisensory inputs from different modalities as well as their sequential encoding thereby enrich the formation of stable memory traces^39,41^. As multivariate approaches have shown that neural pattern reinstatement is indeed associated with episode-specific feature sequences, this study aims to investigate the oscillatory mechanisms underlying the retrieval of sequential information in human memory, focusing on whether the modality sequence in which information was presented during encoding is reinstated during recognition. Specifically, we utilize EEG and MVPA to classify the neural patterns associated with modality sequence reinstatement, providing new insights into the role of oscillatory activity in organizing and retrieving sequential memories across different sensory modalities. Importantly, the current study aims to demonstrate how modality sequence functions as a contextual feature during encoding and recognition (but not temporal reinstatement in the sense of reactivating the sequential order during retrieval; i.e., first visual, second auditory). This design offers a novel perspective on context memory in representing a sharp contrast to previous designs, which employed a parallel presentation of modalities during encoding^71,72^.

## Methods

### Participants

Thirty-six healthy participants were recruited for the experiment. Data from four participants were excluded due to a high number of missing trials (*n* = 1) and poor behavioral performance (*n* = 3). Outliers in terms of memory performance (d’) were identified and excluded if they exceeded ± 3 absolute deviations from the median (MAD;^73^. Therefore, the final sample included *n* = 32 participants (19 females, 52.77% female) with a mean age of 24.25 years (SD = 3.34), ranging from 18 to 33 years. All participants had normal or corrected-to-normal vision and hearing ability and reported no neurological or psychiatric diseases. They gave written informed consent and received financial reimbursement for participating in the study. The Hamburg Medical Council ethics committee (PV5893) approved this investigation. We confirm that all research was performed in accordance with relevant guidelines and regulations.

### Task and procedure

**Fig. 1:**
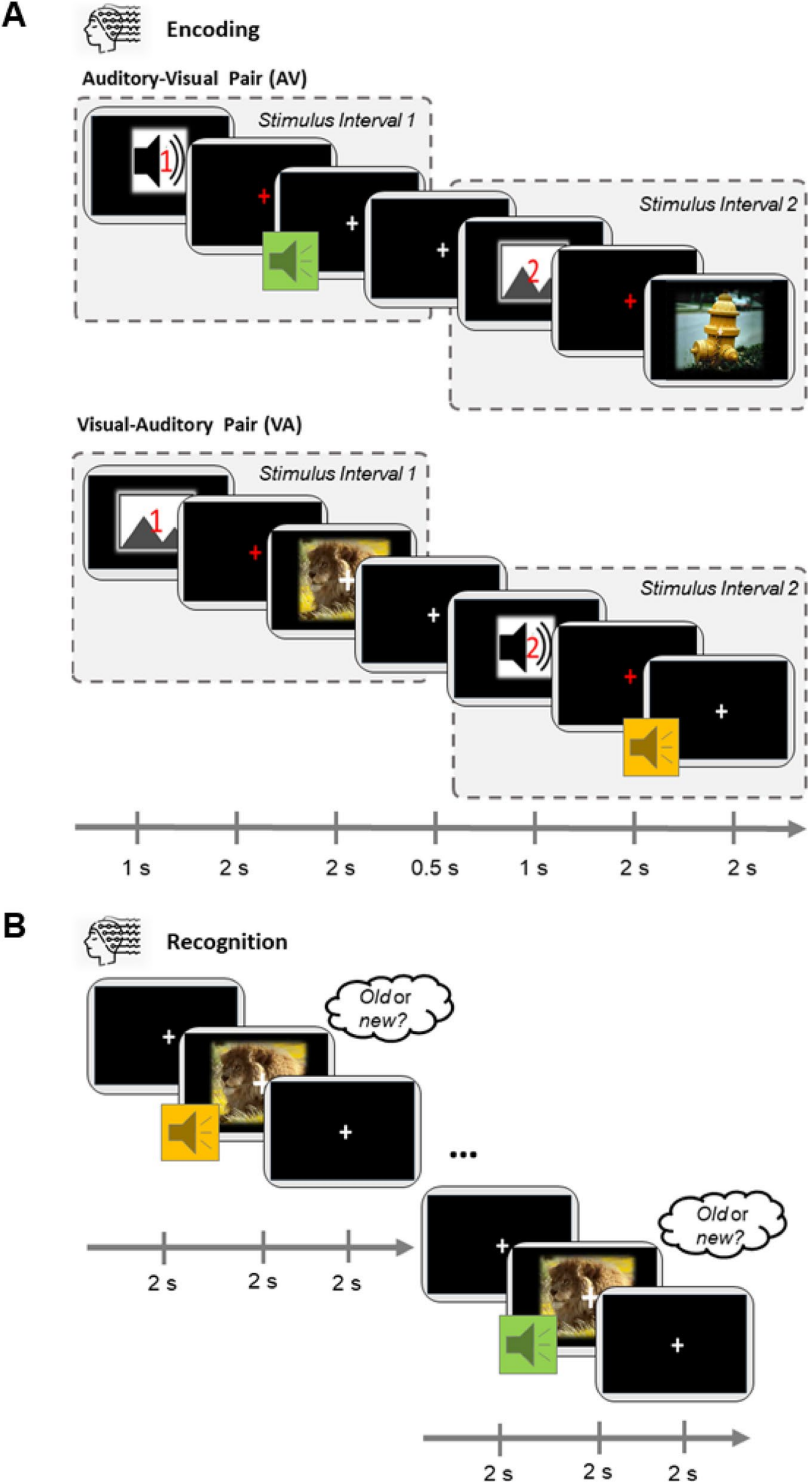
Schematic overview of the encoding task in both sequence variations as well as the following recognition task. (**A**) The two different modality order sequences in the encoding task. Stimuli were presented sequentially. The trial started with a cue, indicating which stimulus modality is presented first followed by a red fixation cross. The red fixation cross indicated that a stimulus would be presented in the next 2 s. Afterwards, a complex natural image was presented. The presentation of the second stimulus of the pair followed the same procedure. We utilized two different encoding sequence orders: either the sound was present first and the image afterwards (auditory-visual; AV-condition or the image was presented first and the sound afterwards (visual-auditory; VA-condition). For the analysis of the encoding task, the task was subdivided into Stimulus Intervals 1 and 2, indicated by the dashed boxes. (**B**) One example trial from the recognition task. Unlike the encoding phase, stimulus pairs were presented simultaneously. Participants had to identify whether the pair **had** been presented during encoding (old) or not (new) within 4 s. The recognition task pairs were composed of the stimuli from the encoding resulting in new and old pairs.

We implemented an explicit sequential associative memory task consisting of an encoding task, a short intermission, and a subsequent recognition task. In order to measure associative multisensory memory, participants were presented sequential image-sound pairs during the encoding task. The images (resolution: 640 × 480 pixels; 24-bit color depth) and sounds (length: 2 s; bitrate: 1411kBit/s) were randomly selected from an internal stimulus database, presenting real-life objects, animals, and landscapes. The individual stimuli were paired pseudo-randomly (semantically congruent stimuli pairs were excluded). Each pair was presented once during the encoding task. In each encoding trial, the stimulus pairs were presented sequentially. Each trial started with a 1-second *modality cue* indicating whether a visual or an auditory stimulus would be presented as the first pair component (Fig. 1). The *modality cue* was represented as an icon, cueing the following modality (image or tone). Additionally, the icon involved a red number, stating whether the next stimulus would be the first or second stimulus of the pair. The *modality cue* was followed by a red fixation cross for 2 s, which served as a visual cue for the upcoming stimulus. Afterwards, the first stimulus was presented, followed by a 500 ms Inter-Stimulus-Interval (ISI). Complementary to the first stimulus presentation, a second *modality cue* indicated whether a visual or an auditory stimulus would be presented as the second stimulus. Again, a red fixation cross served as a visual cue for the upcoming stimulus followed by the announced stimulus and the inter-trial interval (ITI). The ITI was jittered between 3 s and 5 s. Trials were counterbalanced for *modality-order*, such that the encoding task consisted of the same number of visual-auditory (VA) as auditory-visual (AV) trials. We applied a modality cueing procedure, including both modality-specific cues and numerical indicators, to clearly signal the upcoming auditory and visual pairings, thereby facilitating robust and explicit encoding of the stimulus associations. All participants were explicitly instructed to memorize the stimulus pairs, and not to focus on the individual images or sounds. This ensured that subsequent memory would later reflect associative memory, but not item memory. We divided the experiment into three consecutive blocks, each consisting of an encoding and subsequent recognition task. During each encoding task, 47 stimulus pairs were presented. After the encoding task, a short 3-minute intermission followed, during which participants were asked to count down aloud from 100 (115 and 125 in the second and third run, respectively) in steps of 7 (9 and 13 in the second and third run, respectively). In the subsequent recognition task, participants were presented with the 47 stimulus pairs shown during the previous encoding phase, as well as 47 new pairs. Both components of a pair were presented in parallel, in sharp contrast to the encoding task, where the components were shown sequentially. New pairs consisted of the same individual components that rendered the pairs from the encoding task, but were shuffled to create 47 new pairs. The participants were asked to indicate via button press whether the presented pairs were already known from the encoding task or not. Stimulus presentation lasted for 2 s, and no cue was used. The inter-trial interval was fixed to 5 s. The recognition task was followed by a short break of 3 to 5 min, followed by the encoding task of the next run. Across the three blocks, 141 stimulus pairs were presented during encoding, and 282 were presented during recognition in total.

### EEG data acquisition

EEG data were collected using a 64-channel Ag/AgCl active electrode system (ActiCap64; BrainProducts, Gilching, Germany), arranged in accordance with the extended 10–20 system^74^. Sixty electrodes were positioned at the most central scalp locations. To facilitate offline artifact removal, a bidirectional bipolar electrooculogram (EOG) was concurrently recorded using the remaining four electrodes. These bipolar EOG electrode pairs were positioned above and below the left eye, as well as at the lateral ends of the bicanthal plane. FCz served as the reference electrode for data acquisition, while the ground electrode was situated at position Iz. Signals were digitized at a sampling rate of 500 Hz and was amplified with a low cut-off frequency of 0.53 (0.3 s time constant). Impedances were maintained below 10 kΩ throughout the recording session.

### EEG preprocessing and time-frequency decomposition

The acquired EEG data were preprocessed offline using the FieldTrip toolbox^75^ in MATLAB (Release 2022a, The MathWorks Inc., Natick, Massachusetts, USA). For each participant, the encoding and the recognition task were analyzed separately. For the recognition task, epochs were extracted from − 2500 ms to 3500 ms relative to stimulus onset, resulting in a trial duration of 5 s. A high-pass filter at 0.5 Hz was applied to remove extreme low-frequency fluctuations. The data were visually inspected, and trials containing artifacts, such as high-frequency noise indicating muscular activity or spikes resembling poor electrode connections, were removed. Independent Component Analysis (ICA) was used to identify components corresponding to blinks and other ocular activity, and the data were corrected accordingly. On average, 4.8 components (SD = 3.31) were removed per subject. The data were then visually inspected again, and trials with artefacts were excluded. The remaining trials for each subject were split into correct old (remembered; mean = 83.11; SD = 26.40), incorrect old (forgotten; mean = 46.94; SD = 24.08), correctly rejected (mean = 112.46; SD = 17.05) and false alarm (mean = 18.60; SD = 14.78) trials. The correct old trials were additionally split for the “visual-auditory” (VA) and “auditory-visual” (AV) conditions according to modality order in the encoding task, resulting in a similar number of trials in each condition (44 trials on average in each condition; AV: mean = 44.75, SD = 10.79; VA: mean = 44.41, SD = 11.02). Time-frequency decomposition was performed from − 1 to 2 s relative to stimulus onset, covering the 1 to 40 Hz frequency range. This was achieved using a multitaper convolution approach with a sliding Hanning window of 500 ms and a 100 ms step size. Although a 500 ms window has an intrinsic frequency resolution of 2 Hz (1/0.5 s), we applied zero-padding to the maximum trial length, which interpolated the spectrum to a 1 Hz grid, thereby effectively balancing temporal precision with frequency resolution. No baseline correction was applied for the recognition tasks, as the primary focus was on within-subject differences in oscillatory power between the remembered and forgotten pairs. We did not apply baseline correction to preserve potential pre-stimulus effects, which have been implicated in learning and memory processes. Additionally, baseline correction assumes a proportional scaling of oscillatory and non-oscillatory (1/f) activity, an assumption that may not always hold. As a result, the reported effects should be interpreted in terms of relative power changes rather than absolute polarity shifts. Additionally, for the data of the recognition task, the resulting frequency spectra (electrode × frequency × time) from stimulus onset onward, reflecting the processing phase, were used to predict the modality order in the encoding task for each participant and condition. Only trials with successfully remembered items were included in the analysis, while pre-stimulus activity was not included. For the multivariate analyses, the Matlab toolbox for classification and regression of multi-dimensional data (MVPA-Light;^76^ was used. A support vector machine (SVM) with a k = 5-fold cross-validation was used for classification with a five-time repetition. The classifier was trained on all electrodes using single-trial frequency spectra.

The preprocessing routine and the time-frequency analysis for the encoding task were the same as for the recognition task, with deviations during epoching. The deviation in epoching procedure resulted from the difference in presentation mode. While sound-image associations were presented simultaneously in the recognition task, the associations were presented sequentially in the encoding task. Epochs were extracted from the first modality cue until 2 s after the onset of the second stimulus, resulting in a trial duration of 10.5 s. Using ICA, on average 4.8 independent components were rejected from each individual data set (SD = 2.61). After preprocessing, the remaining trials for each subject were split into a “visual-auditory” and “auditory-visual” condition according to modality order in the encoding task, resulting in a similar number of trials in each condition. These trials were then used in the subsequent analyses of later remembered and forgotten trials (55 trials on average in each condition). After time-frequency decomposition, data were averaged separately for remembered and forgotten trials for each participant.

### Statistical analysis

In the recognition task, the percentages of remembered old pairs (hits), correctly rejected new pairs, forgotten old pairs, and falsely remembered new pairs (false alarms) were extracted. In order to index memory performance, we utilized the sensitivity measure d’, which is the difference between the z-transformed hit and false alarm rates^77–79^. A one-sample t-test against zero was conducted to probe associative memory formation with *d’* as dependent variable. Additionally, a repeated-measures ANOVA was used to analyze reaction times during the recognition task, with the within-subject factors *Pairing* (old vs. new) and *Correctness* (correct: remembered or correct rejection vs. incorrect: forgotten or false alarm).

Statistical analysis of the time-frequency EEG data acquired during the recognition phase of the experiment was conducted to explore the potential effect of remembered and forgotten trials within the low-frequency spectrum. This analysis was further differentiated by modality-independent sequence order during encoding, as well as sequential auditory-visual and visual-auditory presentations. Employing a non-parametric permutation testing approach with cluster-based correction for multiple comparisons, as implemented in the FieldTrip toolbox^75^, we statistically compared time-frequency representations corresponding to remembered trials against those of forgotten trials. The cluster-based permutation test defines a cluster as a set of contiguous significant points in a three-dimensional space comprising electrode location (spatial dimension), frequency, and time. A significant point is identified when the test statistic at a specific electrode, frequency, and time point surpasses a predefined threshold (*p* < .01, uncorrected). These significant points are then grouped into clusters based on their adjacency. This means that a cluster can extend across multiple electrodes, frequency bands, and time points, rather than being restricted to a single dimension. We used the electrode neighbourhood structure defined by Fieldtrip function ft_prepare_neighbours to determine adjacency, ensuring that spatially close electrodes are considered neighbors. In the frequency and time dimensions, adjacency is defined by consecutive frequency bins and time points. To be classified as a cluster, an effect needed to span at least two neighbouring electrodes., preventing isolated effects in one electrode from being classified as clusters. Multiple comparison correction was applied using the cluster-based permutation test (*cfg.correctm = ‘cluster’*,* cfg.method = ‘montecarlo’*), which controls the family-wise error rate (FWER). This means that while individual points initially pass a cluster-forming threshold (*p* < .01, uncorrected), the final significance of a cluster is determined via a permutation-based correction (*p* < .05 two-tailed, cluster-corrected). Thus, only clusters that survived this multiple comparison correction are reported as significant. Subsequently, Monte Carlo method was utilized to generate a distribution of t-values^80^.

The main focus of the study was to probe neuronal reinstatement of the stimulus modality order (visual-auditory, auditory-visual) during subsequent recognition via MVPA. To investigate this effect, we employed MVPA in the time-frequency domain of the recognition task. The SVM classifier distinguished between the two different encoding stimulus modality orders (AV/VA) based on the EEG data from the recognition task in which the previously encoded associations were presented simultaneously. To comprehensively capture neural processing during the recognition phase, we applied MVPA to the entire a priori defined dataset, analyzing the full trial period during recognition from 0 to 2 s relative to stimulus onset, across the 1–40 Hz frequency range and all 60 electrodes. Classification accuracy was assessed using a single–subject k = 5-fold cross-validation procedure, ensuring that model training and testing were performed on separate data splits within each subject to reduce overfitting and improve generalization. We conducted the MVPA on remembered trials of the recognition task to classify the two different modality orders in which the stimulus pairs were presented during the encoding task (*Modality Sequence Classifier*). To assess the statistical significance of the accuracy achieved by the *Modality Sequence Classifier*, we conducted a one-sample *t*-test (one-tailed) comparing the overall classifier accuracy against chance-level (50%) The classifier accuracy was determined by averaging the individual classifier performance within the entire analysis window. This methodology enabled us to assess whether the performance of the *Modality Sequence Classifier* significantly exceeds the chance classification level, thus providing insight into the presence of meaningful patterns associated with memory retrieval as opposed to random classification. Subsequently, to probe statistically significant accuracy of the *Modality Sequence Classifier* across the entire analysis window, we performed a cluster-based permutation t-test, comparing the accuracy values from the *Modality Sequence Classifier* to chance level (50%). To further examine frequency-specific effects, accuracy values were averaged within predefined frequency bands: theta (3–7 Hz), alpha (8–13 Hz), low beta (13–21 Hz), high beta (22–32 Hz), beta (13–32 Hz), and gamma (32–40 Hz). We determined the electrode with the highest mean t-value for each band and computed individual mean accuracy values at these electrodes.

Furthermore, a correlational analysis was conducted to examine the relationship between the oscillatory power contrast between remembered and forgotten trials during encoding task and d’ (memory performance) estimates from the recognition task. This analysis is expected to yield valuable insights into multisensory processing during associative learning and memory. Drawing on previous research^81,82^, we expected power differences to correlate with the memory performance within the low-frequency bands during the presentation of the first and second stimuli. Specifically, these power differences were expected to vary according to the modality sequence order (AV vs. VA condition). To accomplish this objective, we employed a non-parametric cluster-based permutation technique for the correlation analysis to control for alpha-error inflation. Here, we calculated the neuronal activity power differences (remembered > forgotten; AV/VA condition) and correlated these with the behavioural measure d’. Cluster-based permutation t-tests were conducted for each time-frequency data point across channels and participants. Significant differences between conditions (*p* < .05) resulted in adjacent data points being grouped into clusters based on temporal, spatial, and spectral criteria.

## Results

### Successful acquisition of sequentially encoded multisensory associative pairs

**Fig. 2:**
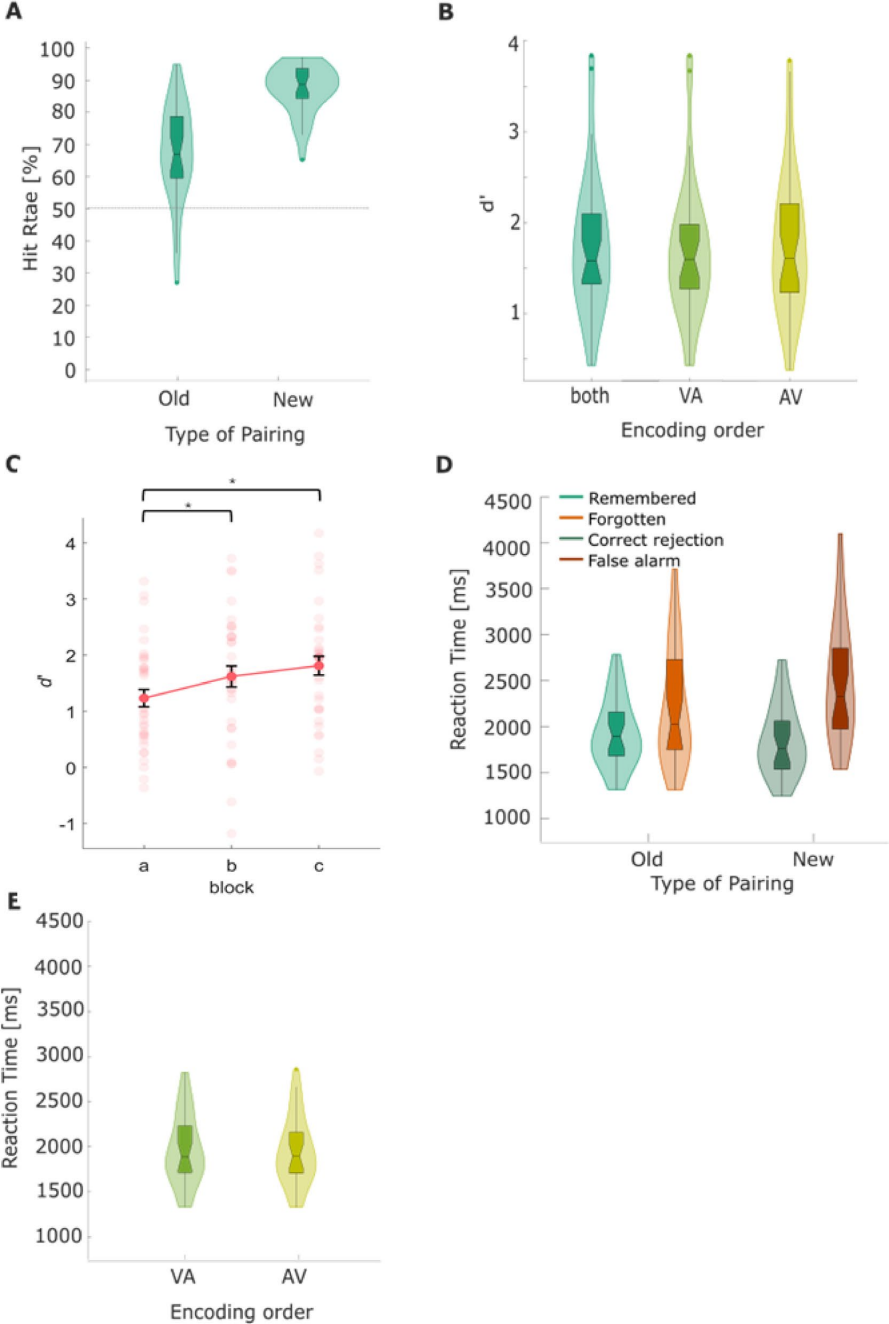
Behavioral results from the recognition task. (**A**) Hit-rate in the recognition task for old and newly rearranged pairs. Within the boxplots, the horizontal lines indicate the median of the subset, while the notch around the median represents its 95% confidence interval. The upper and lower edges indicate quartiles 1 and 3. (**B**) The distribution of memory performance (*d’*) overall, as well as separated between the two modality-order sequences during the encoding task (VA = visual-auditory, AV = auditory-visual). (**C**) The block effect for memory performance (d’). Asterisks indicate significant differences between blocks. *p* < .05(*). (**D**) Distribution of reaction times for the respective response categories from the recognition task, split for remembered, forgotten, correct rejection and false alarm trials. (**E**) Distribution of reaction times for the respective response categories from the recognition task, split between the two modality-order sequences during the encoding task (VA = visual-auditory, AV = auditory-visual) for the remembered trials.

Participants completed one recognition task after each encoding task, which consisted of 47 multisensory associative pairs (Fig. 1). In the recognition task, participants were presented pairs of images and sounds in parallel, which were sequentially presented during previous encoding. This task included previously presented pairs (*old*) and tested memory specificity by presenting newly formed pairs, consisting of old stimuli elements (*new*). Participants had to indicate whether they remembered the presented stimulus pair from the encoding task (old) or whether it was a newly rearranged pair, consisting of an old image with a sound previously paired with another image (new). Overall, participants performed very well in remembering *old* pairs, with an average hit rate of M(SD) = 67.19 (± 15.29%; Fig. 2A). Although the *new* pairs consisted of images and sounds from encoding that were now rearranged, the false alarm rate was low (M(SD) = 12.12 (± 7.68%)). Accordingly, signal detection theory-based analysis confirmed robust learning, expressed by an average associative d’ of M(SD) = 1.76 (± 0.76) independent of stimulus modality order (*t*_(30)_ = 12.69, *p* < .001, *cohen’s d* = 0.59). The d’ estimates of auditory-visual (AV; M(SD) = 1.74 ± 0.76) and visual-auditory stimuli (VA; M(SD) = 1.77 ± 0.77) did not differ significantly (*t*_(30)_ = -0.14, *p* = .884, see Fig. 2B). Taken together, behavioral results confirmed the successful acquisition of sequentially encoded multisensory associative pairs, with no differences in performance due to modality order.

Our study was intentionally designed as an explicit learning and memory paradigm, where participants were specifically instructed to remember the pairs. Given the experimental structure, it was expected that participants adapt their strategies over time, mainly as they were aware that a recognition test followed each block. Crucially, due to the design, these improvements were likely to occur consistently across participants, regardless of their overall performance. Therefore, this effect should not be seen as a systematic bias but rather as an inherent characteristic of explicit learning. To assess whether behavioural performance changed across blocks, we conducted a repeated measures ANOVA on the performance measure d′, which revealed a significant main effect of Block, *F*_(2, 70)_ = 8.92, *p* < .001, *η²* = 0.05. Given that Mauchly’s test indicated a violation of sphericity (*W* = 0.92, *p* = .239), we applied the Greenhouse-Geisser correction (ε = 0.925), yielding a corrected significance value of *p* < .001. Post hoc tests further revealed that performance significantly differed between Block A and Block B (*p* = .025) as well as Block A and Block C (*p* = .002; Fig. 2C).

Next, we compared participants’ reaction times during recognition as an index of memory confidence^83,84^. A two-way repeated-measures ANOVA revealed a significant main effect of the factor Correctness (*F*_(1,31)_ = 69.84, *p* < .001, *η*^*2*^ = 0.69; Fig. 2D), indicating faster responses during remembered pairs, compared to forgotten. Furthermore, this analysis revealed a main effect of Pairing (*F*_(1,31)_ = 9.24, *p* = .005, *η*^*2*^ = 0.23; Fig. 2D), indicating faster responses to old pairs, compared to new. Critically, we observed a significant Pairing × Correctness interaction (*F*_(1,31)_ = 15.56, *p* = .001, *η*^*2*^ = 0.33; see Table 1). The post-hoc *t*-test revealed a significant decrease in reaction times for correctly recognised old pairs (*t*_(126)_ = -0.90, *p* < .001, *Cohen’s d* = − 0.44), indicating an increase in memory confidence in light of correctly retrieved associations compared to forgotten. The reaction time corresponding to auditory-visual (AV; RT = 1944.6 ± 381.7 ms) and visual-auditory stimuli (VA; RT = 1959.4 ± 385.9 ms) did not differ significantly (*t*_*(*62)_ = 0.15, *p* = .877, see Fig. 2E**).** Interestingly, participants correctly rejected new pairs significantly faster compared to mistakenly categorizing them as old (false alarm; *t*_(62)_ = -4.88, *p* < .001), supporting the idea that decision confidence might influence RTs in recognition memory. Also, the response in correctly rejected trials were significantly faster as compared to response in forgotten trials, *t*_(62)_ = -2.84, *p* = .006, suggesting that correctly detecting novel information is easier than failing to recognize old pairs. Responses to forgotten trials did not significantly differ in reaction times as compared to false alarm trials (*t*_(62)_ = -1.63, *p* = .110; Table 2).

**Table 1:** Repeated-Measures ANOVA results for reaction times.

Effect	F(df)	*p*	Partial η^2^
Pairing (Old vs. New)	9.23(1,31)	0.005	0.23
Correctness (Correct vs. Incorrect)	69.84(1,31)	< 0.001	0.69
Pairing × Correctness	15.56(1,31)	< 0.001	0.33

**Table 2:** Post-hoc t-test results for reaction times.

Comparison	t(df)	*p*	*p*_corr_
Remembered vs. forgotten	-2.26(62)	0.027	0.162
Remembered vs. correct rejected	0.81(62)	0.421	1
Remembered vs. false alarm	-4.30(62)	< 0.001***	< 0.001***
Correct rejected vs. forgotten	-2.83(62)	0.006**	0.036*
Correct rejected vs. false alarm	-4.88(62)	< 0.001***	< 0.001***
Forgotten vs. false alarm	-1.63(62)	0.110	0.660

p_corr_ relates to p-values after Bonferroni correction. *p* < .05(*), *p* < .01(**), *p* < .001 (***).

### Successful recognition of multisensory associations relies on alpha/beta oscillations

**Fig. 3:**
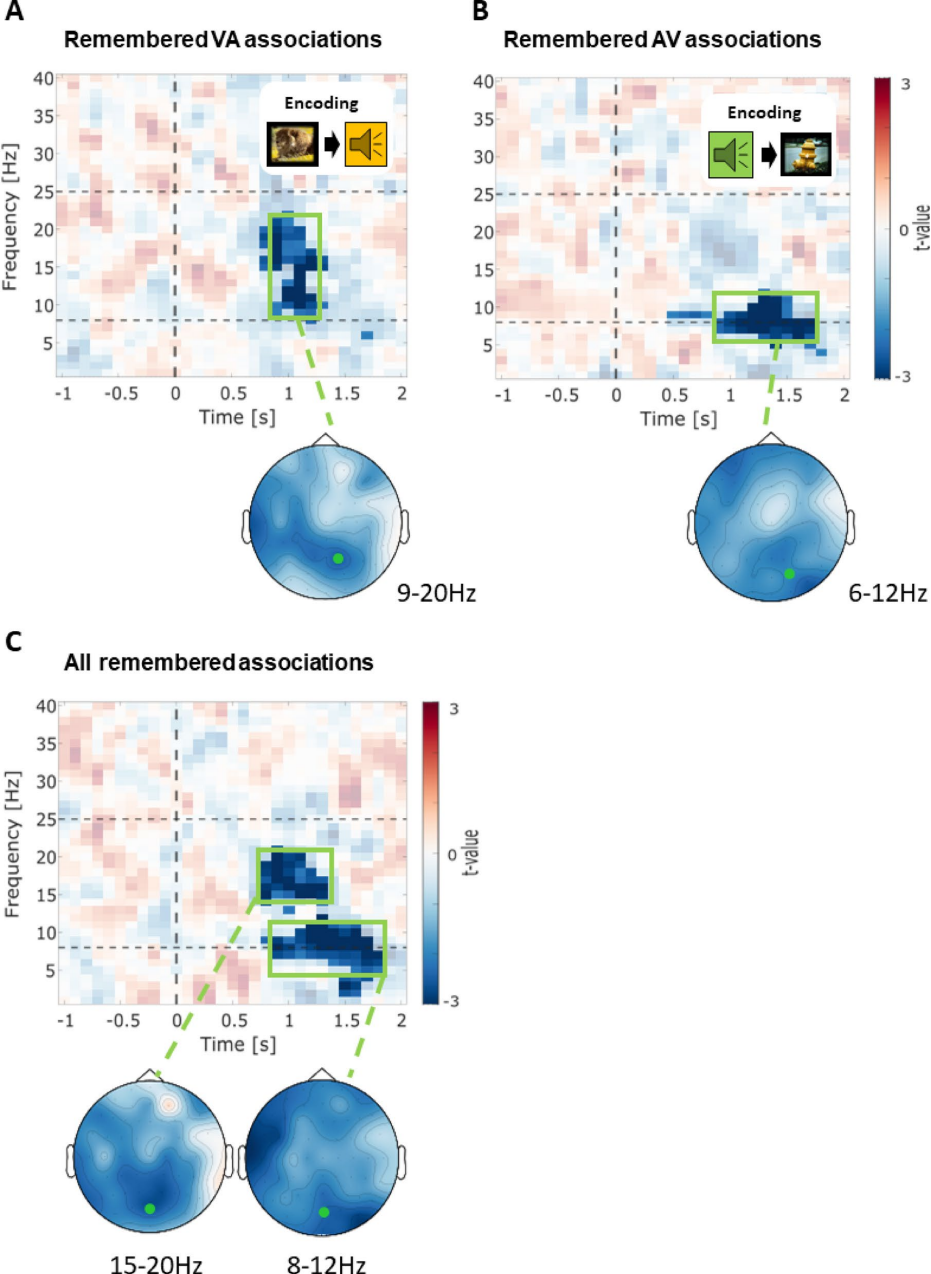
Memory effects on time-frequency power before and during recognition for electrodes with maximal t-value for corresponding encoding stimulus modality order. (**A**) Time-frequency plot of the statistical comparison of REMEMBERED > FORGOTTEN oscillatory power at P2 for recognized associations, which were presented in the visual-auditory sequence during encoding. Opaque data points show a significant difference at *p* < .05 (corrected). The lower panel shows the topographical distribution within the significant cluster during stimulus presentation in the theta-alpha range (0.9 to 1.2 s; left). The green marker illustrates P2. (**B**) Time-frequency plot of the statistical comparison of REMEMBERED > FORGOTTEN oscillatory power at PO4 for recognized associations, which were presented in the auditory-visual sequence during encoding. Opaque data points show a significant difference at *p* < .05 (corrected). The lower panel shows the topographical distribution within the significant cluster during stimulus in the alpha/beta range (0.8 to 1.4 s). The green marker illustrates P2. (**C**) Top: Time-frequency plot of the statistical comparison of REMEMBERED > FORGOTTEN oscillatory power at POz, independent of modality order presentation during encoding. The vertical line marks the stimulus onset, and the horizontal lines mark the frequency bins alpha and beta (8 Hz and 25 Hz). Opaque data points show a significant difference at *p* < 0. 5 (corrected). Negative t-values signify higher power in FORGOTTEN trials. The lower panel shows the topographical distribution within the significant cluster during stimulus in the beta range (0.8 to 1.3 s; left) and the significant cluster in the alpha range (0.9 to 1.8 s, right). The marker illustrates POz.

In the next step, we investigated the oscillatory power differences between remembered and forgotten multisensory pairs within the recognition task, which presented the previously sequentially encoded pairs simultaneously. Here, participants had to indicate whether the presented pair was previously shown in sequential order during encoding. In the following analysis, we split the recognition trials according to their modality-sequence during encoding (VA/AV), and conducted time-frequency analyses. In VA associations (remembered > forgotten) we observed a significant negative cluster of oscillatory activity covering the high theta to low beta frequency range (0.8–1.2 s after stimulus onset; 7–23 Hz; negative cluster: *p* < .003, SD = 0.001). This indicates that remembered pairs, which were represented in a VA sequence order during encoding, were associated with alpha and beta power during memory retrieval as compared to forgotten pairs. This effect was primarily driven by activity differences in frontotemporal and lateral-occipital areas (0.8–1.2 s after stimulus onset, 7–23 Hz; Fig. 3A). Interestingly, AV trials showed a different pattern, including a negative cluster in the theta and alpha range (0.5–1.9 s; 6–13 Hz; negative **c**luster 1: *p* < .002, SD = 0.009; negative **c**luster 2: *p* < .045, SD = 0.005), indicating that remembered pairs, which were represented in an AV sequence order during encoding, were associated with theta and alpha power during memory retrieval as compared to forgotten pairs. This effect was primarily driven by activity differences in parietal-occipital areas (0.5–1.9 s after stimulus onset, 6–13 Hz; Fig. 3B). The results indicate differential processes concerning the oscillatory processing of modality-sequences during recognition and were used to restrict the following MVPA analysis. When analyzing both conditions (combining AV and VA) together as an independent modality-sequence condition, we observed similar significant neuronal activity effects, further reinforcing the underlying processing patterns across modalities. The results revealed a negative cluster covering the theta (median cluster size = 10), alpha (median cluster size = 27), and beta (median cluster size = 18) bands, occurring 0.9 to 1.8 s after stimulus onset (negative cluster: *p* < .001, SD = 0.004; see Fig. 3C). This indicates that remembered pairs were associated with lower theta, alpha, and beta power during memory retrieval as compared to forgotten pairs. This effect was primarily driven by activity differences in frontotemporal and lateral-central areas (1.0 to 1.7 s after stimulus onset, 3–7 Hz) and in lateral parietal regions (0.9 to 1.7 s after stimulus onset, 8–18 Hz; see Fig. 3C).

### Modality-sequences are reinstated as context-features during recognition

The neuronal signature within the recognition task indicated differential processing of the stimulus modality order, which we hypothesized to also be expressed as context-specific features of the underlying memory trace (i.e. the temporal sequence of the stimulus from different modalities). Accordingly, our main hypothesis stated that the neural signature during the recognition task would reflect the modality sequence in which the pairs were presented during the encoding task. To probe this effect, we employed MVPA, moving beyond the univariate comparisons of oscillatory power. The *Modality Sequence Classifier* distinguished between the two different encoding stimulus modality orders (AV/VA) based on the EEG data from the recognition task in which the previously encoded associations were presented simultaneously. Classification accuracy was assessed using a cross-validation (k = 5) procedure. The overall mean classifier performance for the *Modality Sequence Classifier* was 52.26%, which significantly exceeded the chance level of 50% (*t*_(31)_ = 4.28, *p* < .001, *cohen’s d* = 0.76).

For the more detailed classification analyses, we calculated the classification accuracy over all electrodes for each frequency band within the significant data points, as well as the average classification accuracy for the electrode with the maximum mean *t*-value of the entire time within the specific frequency range. The cluster-based permutation test comparing MVPA accuracy values for remembered trials when decoding the two modality orders against chance level (50%) revealed eight significant positive clusters (most prominent cluster: *p* < .001, cluster-level statistic *t* = 29.069, SD = 0.0003, CI range = 0.0006). This cluster encompassed 45.302 data points out of a total of 50.400 (60 channels × 40 frequency × 21 time), covering approximately 89.88% of the analyzed search space. This significant cluster extended from stimulus onset to 2 s post-stimulus, spanning frequencies from 1 to 40 Hz, and was distributed across all electrodes, with the lowest representation in P7 (84.28%) and the highest in FC4 (93.69%). The remaining clusters had *p-*values ranging from 0.017 to 0.047, with their respective cluster statistics and confidence intervals indicating robust effects across multiple frequency bands and time points. No significant negative clusters were detected.

To further interpret the decoding performance, average accuracy values were computed over the significant time points identified by the cluster-based permutation test, focusing on time points and electrodes with the highest mean *t-*values. To identify the electrodes exhibiting the strongest effects, we computed the mean *t*-value for each electrode by averaging all significant *t*-values (*p* < .05) across frequency bands and time points. This metric served as an index of the relative effect size at each electrode, highlighting regions that consistently demonstrated robust neural discrimination effects in the cluster-based permutation test. By focusing on electrodes with the highest mean *t*-values, we aimed to characterize the spatial distribution of the most pronounced neural decoding effects during the recognition task. The overall mean accuracy across all frequencies (1–40 Hz) was 52.26%, with the highest mean *t-*value observed at electrode F4 (*t* = 4.18). The mean individual accuracy at this electrode was 52.32%, while the highest individual accuracy value reached 62.38% at 0.8 s and 30 Hz. The maximal *t-*value was reached at F4 (*t* = 6.58).

When examining specific frequency bands, the theta range (3–7 Hz) yielded the highest mean accuracy of 52.49%, with the strongest effect at electrode AF7 (*t* = 4.22), and an individual peak accuracy of 58.80% at 0.4 s and 7 Hz. The maximal *t*-value was reached at AF7 (*t* = 6.29). In the alpha band (8–13 Hz), the mean accuracy was 52.40%, with the highest *t*-value recorded at electrode Fpz (*t* = 3.92), and an individual maximum accuracy of 58.67% at 0.1 s and 11 Hz. The maximal *t*-value was reached at F4 (*t* = 6.58). The low beta range (13–21 Hz) showed a mean accuracy of 52.29%, with the most significant effect at electrode Fpz (*t* = 3.80) and a peak accuracy of 58.63% at 0.4 s and 21 Hz. The maximal *t*-value was reached at FC2 (*t* = 5.93). Similarly, the high beta range (22–32 Hz) demonstrated a mean accuracy of 52.22%, with the strongest effect at electrode P1 (*t* = 4.27) and a peak accuracy of 58.29% at 1.9 s and 24 Hz. The maximal *t*-value was reached at CP2 (*t* = 6.31).

When considering the full beta range (13–32 Hz), the mean accuracy was 52.24%, with the highest *t*-value at P1 (*t* = 4.07) and an individual maximum accuracy of 61.00% at 1.9 s and 24 Hz. The maximal *t-*value was reached at CP2 (*t* = 6.31). The mean accuracy in the gamma band (32–40 Hz) was 52.23%, with the effect at electrode P2 (*t* = 4.69) and an individual peak accuracy of 52.98% at 0.1 s and 33 Hz. The maximal t-value was reached at PO8 (*t* = 6.18). All results are summarised in Table 3. These findings suggest that decoding performance was significantly above chance level across multiple time points, frequencies, and electrode locations, with particularly strong effects in the theta, alpha, and beta bands.

**Table 3:** Average Accuracy for each frequency range and at the electrode with the maximummean t-value.

Frequency range [Hz]	Mean Accuracy [%]	Peak mean t-value Electrode	Time [s]	Frequency [Hz]	Peak mean t-value Max. [%]
Overall [1 40]	52.26	F4	0.8	30	62.38
Theta [3 7]	52.49	AF7	0.4	7	58.80
Alpha [8 13]	52.40	Fpz	0.1	11	58.67
Beta [13 21]	52.29	Fpz	0.4	21	58.63
Beta [22 32]	52.22	P1	1.9	24	58.29
Beta [13 32]	52.24	P1	1.9	24	61.00
Gamma [32 40]	52.23	P2	0.1	33	52.98

**Fig. 4:**
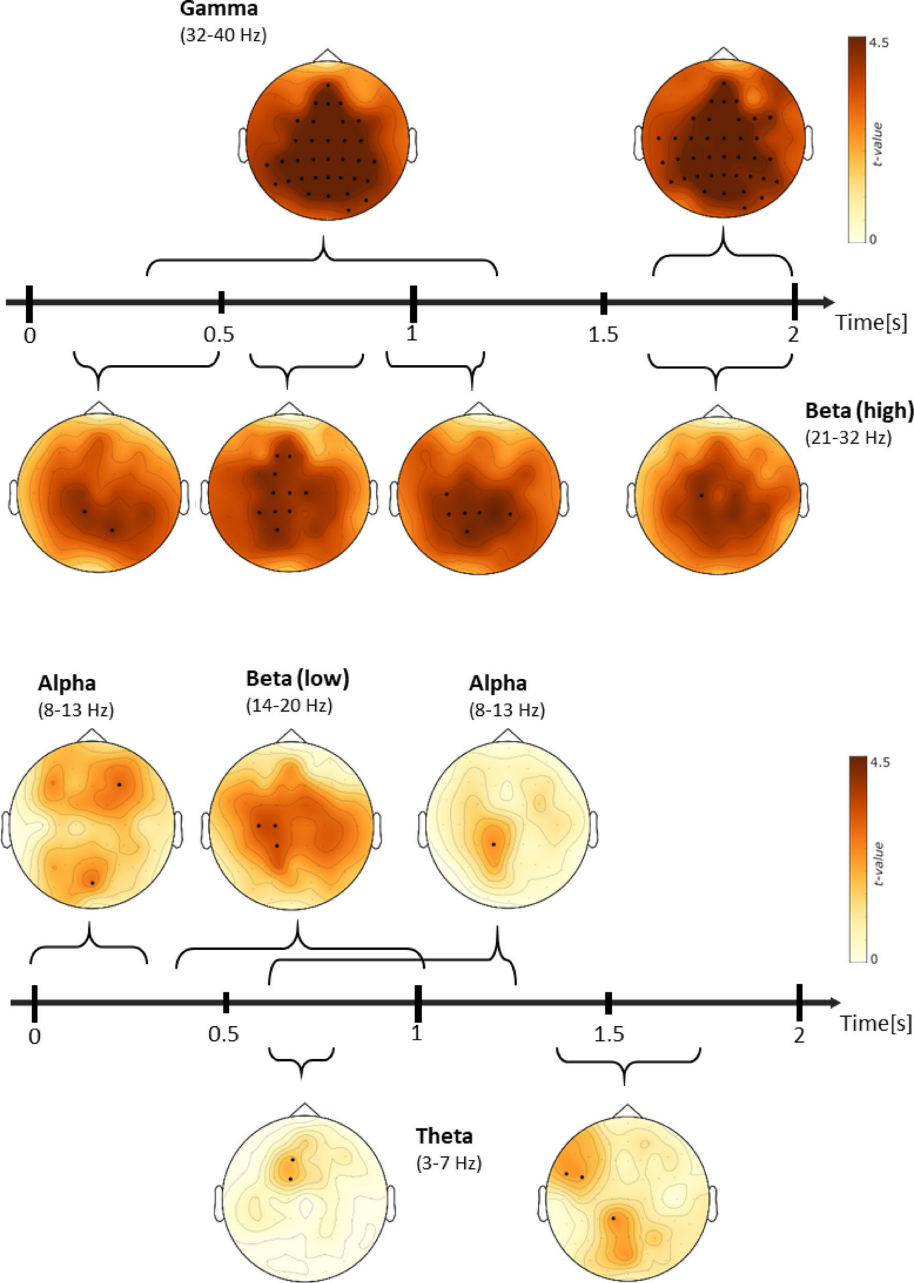
Topographical plots of *t*-values from the cluster-based permutation test comparing MVPA accuracy for remembered trials against the chance level of 50%. The plots illustrate the distribution of significant effects across the scalp for different frequency bands (theta, alpha, low beta, high beta, and gamma) over the trial time course. Highlighted electrodes indicate regions with the highest number of neighbouring significant data points), reflecting areas with the strongest decoding effects. The gamma band showed significant effects between 400–1300 ms and 1700–2000 ms, with strong activations in centro-posterior regions. The high beta band displayed significant clusters in multiple time windows (100–500 ms, 600–800 ms, 900–1200 ms, and 1700–2000 ms), predominantly in centro-parietal areas. The low beta band exhibited significant effects between 400–1000 ms, mainly over central electrodes. The alpha band showed spatially distributed effects in early (0–300 ms) and later (600–1300 ms) time windows, particularly in partial-occipital and fronto-central sites. The theta band revealed significant clusters in two discrete time windows (600–700 ms and 1400–1700 ms), with the strongest effects observed in frontal and central regions. The results suggest that significant decoding effects are not uniformly distributed across the scalp but are concentrated in specific electrode regions, particularly in central and parietal areas in the beta and gamma range and frontal areas in the theta and alpha range.

**Fig. 5:**
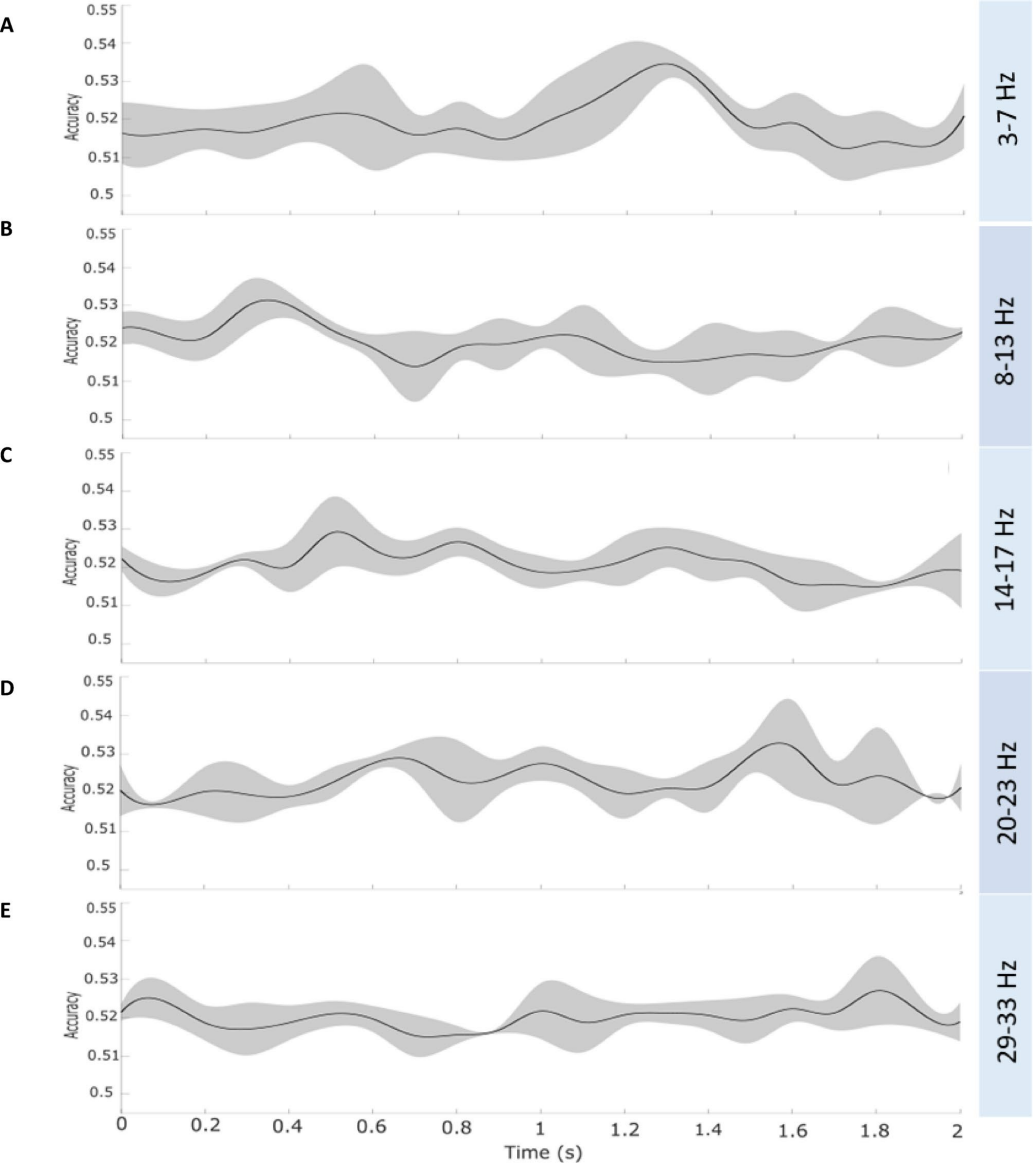
Full-time courses of accuracy values from exemplary electrodes for each frequency range. Time-resolved decoding accuracy (MVPA) for remembered trials to classify modality order from 0 to 2 s relative to stimulus onset is shown at exemplary electrodes. Accuracy values, averaged within the respective frequency bands, range from 51–54%. (**A**) Theta (3–7 Hz) at Cz exhibits a subtle peak around 1.3 s. (**B**) Alpha (8–13 Hz) at F4 shows a slight peak around 0.3 s. (**C**) Decoding accuracy in the low beta (14–17 Hz) at Fz, (**D**) high beta (20–23 Hz) at C3, and (**E**) gamma (29–33 Hz) at F4 bands fluctuates over time but did not reveal distinct classification peaks. Caution should be exercised, as the averaging of accuracy estimates over multiple time points and across participants may have contributed to an overall reduction in classification performance.

To further explore the spatial distribution of significant effects, we visualized topographical maps of *t-*values obtained from the cluster-based permutation test for different frequency bands (theta, alpha, low beta, high beta, and gamma) over the trial time course (Fig. 4). Interestingly, the higher beta and gamma frequency range (21–40 Hz) exhibited pronounced discriminative power between the two conditions during the entire stimulus presentation over the centro-parietal electrodes, specifically strongest at the beginning (0.4 to 1.3 s) and the end (1.7 to 2.0 s). Additionally, also early (0 to 1.0 s to stimulus onset) centro-frontal electrode cluster in the lower frequency range (3 to 20 Hz) showed a significant classification performance. The results demonstrate a convergence between the neural activity patterns during successful recognition and retrospective discrimination between the modality order of the sequential encoding during recognition. In sum, these findings highlight the ability to encode and differentiate VA from AV sequences during the retrieval task, as evidenced by the distinct neural patterns observed in the EEG data during subsequent recognition. In addition to the averaged accuracy values from electrodes with the highest mean *t*-values, time-resolved decoding accuracy (MVPA) for remembered trials is shown at exemplary electrodes across different frequency bands (Fig. 5).

### Successful recognition of multisensory associations relies on low-frequency oscillations during encoding

**Fig. 6:**
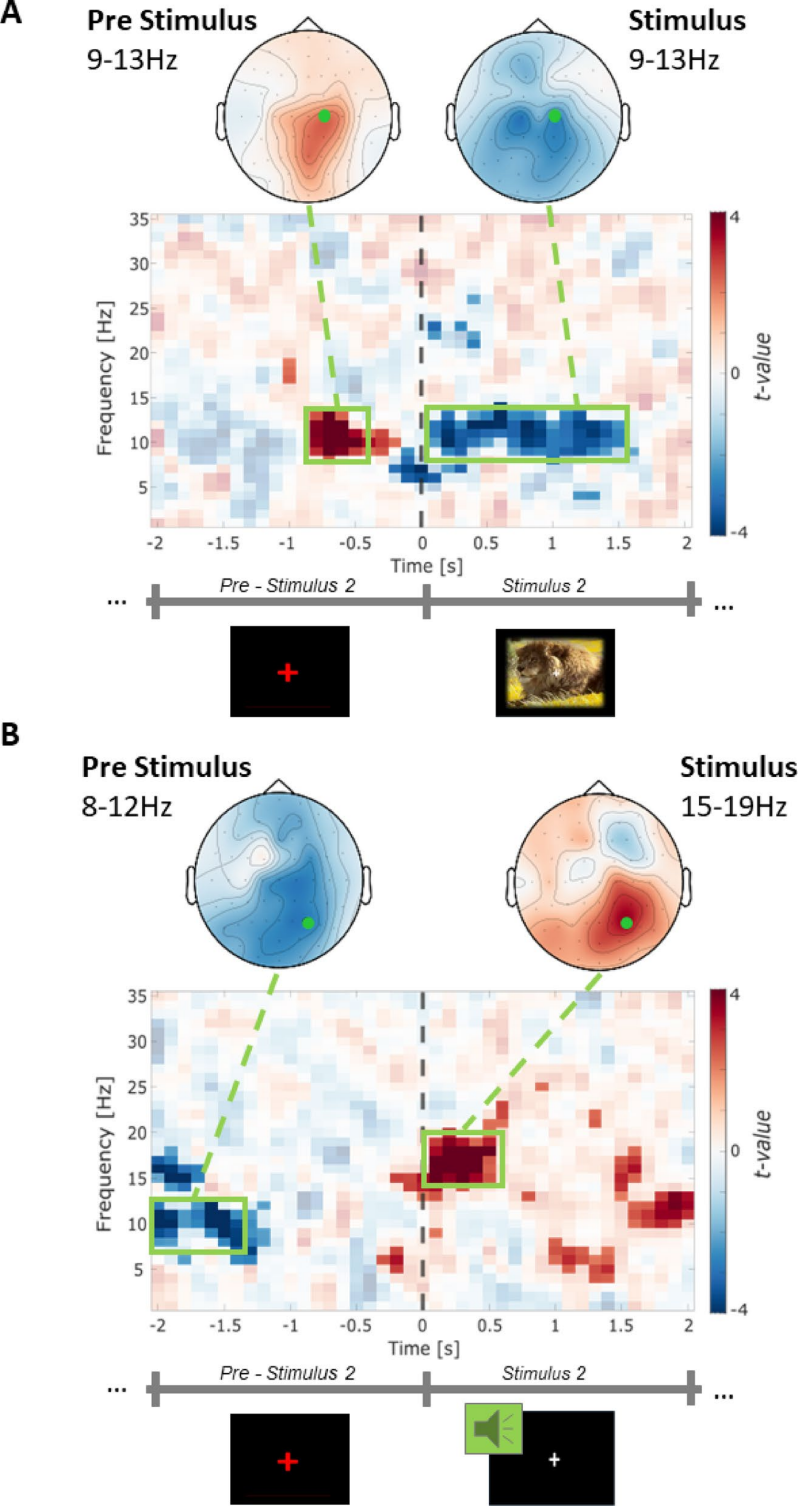
Correlation between the power difference of remembered vs forgotten trials in Stimulus interval 2 and the memory performance (d’) from the recognition task. (**A**) The correlation between the power differences and the memory performance as measured by d’ during visual stimuli presentation (AV pair) at C2. Topographical distribution within the significant cluster in the pre stimulus (-0.8 to -0.1 s; left) and the significant cluster in the stimulus presentation (0 to 1.5 s) in the alpha range (9 to 13 Hz, right) during visual stimuli presentation. The marker illustrates C2. (**B**) The correlation between the power differences and the memory performance as measured by d’ during auditory stimulus presentation (VA pair) at P4. Topographical distributions are shown for the pre-stimulus cluster (-2 to -1.3 s; 8–12 Hz) and stimulus presentation cluster (0–0.5 s; 15–19 Hz). The green marker illustrates P4.

As an explorative analysis, we analyzed the dynamics between the neuronal activity from the encoding task and the recognition performance. Thus, we asked whether specific oscillations during encoding propel successful memory formation. First, we computed the differences in oscillatory power between later remembered and forgotten trials separately for the presentation of the first and second stimulus of each pair. Therefore, we focused on the 2-s period before and after each stimulus onset for the VA and AV conditions, resulting in two analysis time windows (Stimulus Interval 1 and Stimulus Interval 2; Fig. 1). Initially, we analyzed oscillatory data independent of the stimulus modality order, dividing Stimulus intervals 1 (SI1) and 2 (SI2). The differential time-frequency spectra between remembered and forgotten pairs were computed in each participant. These difference values were correlated with associative d’ values from the recognition task. Results revealed significant correlations within SI2 for both encoding sequence conditions. During the visual stimulus of AV pairs, two clusters of significant negative correlation between associative d’ and average power differences (remembered > forgotten) were revealed at multiple electrodes in the parietal and central region in the alpha band (pre-stimulus positive cluster: *p* < .042, SD < 0.005; post-stimulus negative cluster: *p* < .013, SD = 0.003, Fig. 6A) at multiple electrodes in the central-parietal region (Fig. 6A) within the alpha range. In VA pairs, however, during the presentation of the auditory stimulus, we observed a significant positive post-stimulus correlation (*p* < .006, SD = 0.002; Fig. 6B) and negative pre-stimulus correlation (*p* < .018, SD = 0.003) at multiple electrodes in the parietal-occipital region (Fig. 6B) within the alpha and beta range.

## Discussion

Successful retrieval of events is strongly bound to the context of encoding^55,85,86^. While it is well established that context can be reflected as the surrounding environment^31,87,88^, it can also be represented differentially, i.e. as the sequence of episode-specific features. In line, context feature reinstatement (of sequential information) has been shown to be of central importance for memory encoding and retrieval processes^51,88–91^. The role of contextual reinstatement of multisensory features that stem, i.e. from the auditory and visual domain, remains so far unexplored. Here we aimed to shed light on the oscillatory mechanisms underlying the recognition of sequentially encoded multisensory episodes. Our findings show that modality sequences are (incidentally) encoded within the memory trace and serve as a context feature that drives recognition based on theta, alpha and beta frequency pattern reinstatement.

It is well established that context reinstatement plays a crucial role in memory retrieval, allowing the brain to access the temporal and environmental cues associated with past events^51,52^. However, previous research has largely focused on unimodal stimuli, rendering our understanding about how complex multisensory sequences are encoded and retrieved incomplete. In the current study, participants acquired and retrieved the image-sound/sound-image associations overall very well, with recognition performance as measured by d’ being comparable to similar study designs^72,92–94^. Importantly, recognition performance did not differ when comparing AV (auditory-visual) vs. VA (visual-auditory) pairs, suggesting that the order of features itself did not influence memory formation. While we did not test for incidental stimulus acquisition, several studies report reinstatement of encoding specific features in memory tasks^29,95,96^.

MVPA of EEG data revealed distinct neural signatures depending on the modality sequence presented during encoding, even though overall memory performance for both conditions was the same. This suggests that the brain encodes the order of multisensory episodes as part of the contextual memory trace, which aids in the retrieval process. This finding is consistent with prior work demonstrating context-specific temporal patterns during both encoding and retrieval processes^97^. Furthermore, it aligns with the Context Maintenance and Retrieval (CMR) model, which states that temporal and contextual features of episodes are essential components of the memory trace^52^. Our study extends this model by showing that modality order, as a contextual feature, can be decoded from oscillatory activity during memory retrieval​.

Our multivariate results from EEG recordings suggest that decoding performance went significantly above chance level across multiple time points, frequencies, and electrode locations, with particularly strong effects in the theta, alpha, and beta bands. Theta oscillations have been associated with the binding of information into coherent memory traces and are crucial for organizing sequentially ordered working memory items^60,68,98^. These oscillations are thought to coordinate neural activity across different brain regions, facilitating the binding of sensory inputs into a coherent memory trace^97–99^, acting as the “glue”^100^. Alpha oscillations have been generally related to the inhibition of irrelevant information and are involved in processing incoming information relevant to memory^101,102^. Decreases in alpha power during memory tasks have been associated with enhanced memory performance, particularly in semantic encoding tasks^103,104^, while beta oscillations have been linked to memory formation, with elevated pre-stimulus beta power associated with successful memory encoding^105,106^. This activity is thought to reflect a memory-promoting state, possibly moderated by attentional or inhibitory processes^105^. In sum, theta, alpha, and beta oscillations play distinct yet interconnected roles in memory processing^107,108^. Taken together, the fact that the MVPA analysis revealed significant classification of modality order across different oscillatory bands provides strong evidence that sequential information in the form of a context feature is retained and reinstated during retrieval rather than merely reflecting general associative activation. Thus, our results suggest that encoding processes are sequence-specific, with VA pairs potentially engaging greater anticipatory processing due to the nature of auditory stimulus processing. One may speculate, that the reinstatement of the observed oscillatory patterns may facilitate the synchronization of neural activity across sensory processing regions, ensuring that the original modality sequence is represented during recognition within the specific memory trace, which has been suggested by several human studies^4,11,82,109,110^.

Considering the potential cognitive processes elicited by the experimental design, one could argue that the initial stimulus might evoke visual or auditory mental imagery, creating an expectation of the second stimulus. In fact, there is evidence showing that mental imagery could also influence associative memory retrieval by engaging both modality-specific and modality-independent neural networks^111,112^, thereby aiding in overcoming potential modality mismatches during encoding induced by incongruent association pairs^113^. This process involves the activation of sensory-specific regions, such as the visual and auditory cortices, alongside a modality-independent core network, including the default mode network, which supports imagery across different sensory domains^111,112,114^. The overlap between brain regions involved in mental imagery and those supporting retrieval suggests that successful retrieval relies on the same neural mechanisms that facilitate imagery^111,115^. Furthermore, encoding specificity plays a critical role in remembering, as the reactivation of encoding-related neural patterns benefits retrieval when there is a match between encoding and retrieval modalities but can impair memory under mismatch conditions^116^. However, individuals can flexibly employ mental imagery to compensate for mismatches, generating and maintaining mental representations even when encoding involves incongruent audiovisual information^117^. Moreover, imagery-based strategies, such as integrating items into interactive mental images, have been shown to enhance associative memory, emphasizing the functional significance of mental imagery in retrieval processes^118^.

While these findings demonstrate that mental imagery is connected to memory encoding and retrieval, prior research also suggests that multisensory, sequential encoding can enhance memory through encoding variability, introducing competition effects that alter retrieval dynamics^28,29^. This interpretation gains support from our univariate oscillatory findings, suggesting a modality-specific influence of alpha and beta oscillations during the encoding of sequentially presented audiovisual stimuli. Specifically, for auditory-visual (AV) pairs, increased pre-stimulus alpha and beta power before the visual stimulus and a subsequent decrease during stimulus presentation may indicate a preparatory state followed by active sensory processing. In contrast, the pattern is reversed for visual-auditory (VA) pairs: pre-stimulus decreases in alpha and beta before the auditory stimulus, and increased power during its presentation suggests a shift in processing demands between modalities. Previous research has linked decreased alpha-band activity in the prefrontal and occipital cortex to successful visual encoding, indicating that lower alpha power facilitates visual information processing and enhances memory formation^119–122^. Similarly, increased pre-stimulus beta power has been associated with improved memory formation, potentially reflecting attentional or inhibitory processes that aid in binding stimulus components^105,123^. The observed pre-stimulus increases in beta power in AV pairs may, therefore, indicate an anticipatory mechanism supporting visual encoding, whereas the decrease in VA pairs might reflect a shift in sensory dominance from vision to audition. Alpha and beta oscillations have further been implicated in the processing of expectations and prediction errors. An alpha-to-beta desynchronization (ERD) has been linked to expected stimulus valence, suggesting that these frequency bands contribute to prediction mechanisms that influence encoding efficiency^124^. In audiovisual tasks, alpha oscillations modulate sensory processing and attention, influencing the temporal integration of stimuli^125^. However, alpha activity does not solely predict auditory stimulus detection consistently due to its interaction with broadband neural activity^126^. Beta oscillations, on the other hand, are associated with top-down control processes and enhance memory formation across sensory modalities, including auditory processing^105^. Taken together, these findings suggest that alpha and beta oscillations in sequential encoding are modality-dependent rather than purely memory-driven. The observed pre-stimulus shifts in power may reflect preparatory mechanisms that optimize encoding by modulating attention and sensory processing demands across modalities. Specifically, lower alpha power in occipital-parietal regions has been associated with improved perceptual sensitivity and the enhancement of stimulus representations^127^, supporting the idea that modality-dependent oscillatory changes may reflect the differential engagement of sensory and integrative processes during retrieval. Finally, theta oscillations play a critical role in cross-modal binding, supporting associative memory by synchronizing neural activity across sensory regions^128^. Given the angular gyrus’ role in multimodal integration^129^ and the contribution of multisensory cues to episodic retrieval^130–132^, it is likely that power changes across frequency bands reflect both modality-specific processing and memory-related mechanisms in an interactive manner. Thus, the observed oscillatory dynamics align with well-established mechanisms of sensory reactivation, cortical excitability, and associative memory retrieval.

The role of modality order in retrieval is further supported by the finding that above-chance decoding of modality order from EEG activity during retrieval indicates its integration into the memory trace. If one modality had dominated encoding, successful decoding of modality order would not be expected. Prior research supports the idea that encoding modality order contributes to retrieval by providing structured cues that facilitate reconstruction of past experiences^133^. Contextual information is a well-established component of episodic memory, with hippocampal mechanisms playing a crucial role in binding sensory details into coherent memory representations^22,85,103,134,135^. Our study extends this body of work by demonstrating that modality order, as a contextual feature, can be decoded from oscillatory activity during retrieval. While our data do not conclusively establish a causal link between modality order encoding and retrieval success, they provide novel insights into the neural dynamics supporting multisensory sequential memory. Although no behavioural data confirm explicit retrieval of order information, the significant decoding results indicate that modality order was included in the memory trace and reinstated during retrieval. Future studies should explore the extent to which such reinstatement contributes to explicit order memory and whether implicit representations influence retrieval performance.

Finally, we observed modality effects in parietal locations during both, encoding, and recognition tasks. This might reflect multisensory association processes^136,137^, as the parietal cortex is crucial for integrating information from various sensory modalities^138–141^. Interestingly, our findings align well with recent work around multisensory processing, which shows that information from different sensory modalities is integrated within several cortical regions (e.g. the parietal lobe;^139,141,142^**)**. While the intraparietal sulcus (IPS) is known to process multisensory information^143–145^, the angular gyrus has been shown to be centrally involved in binding information into coherent narratives^131^. In our results, classifier accuracy derived from MVPA was highest at centro-parietal electrodes (e.g., CP1), covering the superior parietal lobe. This might suggest a distinct role of the superior parietal cortex in multisensory sequential reinstatement processes, which supports the hypothesis that the neural systems for sequence encoding and multisensory integration are closely linked to facilitate the binding of presented items, thereby forming an episode.

In conclusion, our study provides new insights into the neural mechanisms underlying multisensory memory retrieval. The findings of this study yield important implications for our understanding of memory processes. First, the (incidental) encoding of modality sequence order as a context feature suggests that the brain actively integrates temporal and sensory information during memory formation. This has important implications for models of episodic memory, particularly those that emphasize the role of context^52^. Our findings suggest that the neural mechanisms underlying context-feature retrieval are not limited to unimodal tasks^4,51,64,146^. Instead, they extend to more complex multisensory episodes, during which we encode and integrate sequences of different sensory modalities. Crucially, the sequence of modalities as a contextual feature within the memory trace directly affects memory retrieval, cognitive control, and learning processes^147–150^. Prior research suggests that different sensory modalities contribute uniquely to encoding and retrieval mechanisms, with auditory and visual sequences influencing attentional engagement and memory consolidation in distinct ways (e.g.,^151,152^. Auditory sequences, for instance, have been linked to more durable temporal structuring, while visual sequences often benefit from spatial organization^71^. Recognizing modality sequence as a contextual feature allows us to investigate how the structure of sensory input shapes memory representations rather than focusing solely on content-based associations. Understanding these effects can help optimize learning and memory strategies by leveraging the strengths of different modalities. This might offer insights into educational and rehabilitative applications where multimodal integration plays a key role. Our findings provide new insights into how the brain encodes and retrieves complex episodic memories, particularly those that involve multisensory information.

## Data Availability

The raw EEG and behavioral data underlying our findings have been uploaded to an open repository (https://www.fdr.uni-hamburg.de/; https://www.fdr.uni-hamburg.de/record/17120) for accessibility.
